# Risk perception and terrestriality in primates: A quasi‐experiment through habituation of chacma baboons (*Papio ursinus*) in Gorongosa National Park, Mozambique

**DOI:** 10.1002/ajpa.24567

**Published:** 2022-06-14

**Authors:** Philippa Hammond, Lynn Lewis‐Bevan, Dora Biro, Susana Carvalho

**Affiliations:** ^1^ Primate Models for Behavioural Evolution Lab, School of Anthropology and Museum Ethnography University of Oxford Oxford UK; ^2^ Department of Zoology University of Oxford Oxford UK; ^3^ Department of Brain and Cognitive Sciences University of Rochester Rochester New York USA; ^4^ Paleo‐Primate Project Gorongosa National Park Gorongosa Sofala Mozambique; ^5^ Interdisciplinary Center for Archaeology and Evolution of Human Behaviour (ICArEHB) Universidade do Algarve Faro Portugal

**Keywords:** baboons, landscape of fear, primate habituation, risk perception, terrestriality

## Abstract

**Objectives:**

Habituation is a common pre‐requisite for studying noncaptive primates. Details and quantitative reporting on this process are often overlooked but are useful for measuring human impact on animal behavior, especially when comparing studies across time or sites. During habituation, perceived risk of a stimulus—human observers—is assumed to decline with repeated exposure to that stimulus. We use habituation as a quasi‐experiment to study the landscape of fear, exploring relationships between actual risk, perceived risk, mediating environmental variables, and behavioral correlates.

**Materials and Methods:**

We recorded vocalizations and observer‐directed vigilance as indicators of perceived risk during habituation of two troops of chacma baboons (*Papio ursinus*) in Gorongosa National Park, Mozambique. Here, we model changes in these variables as a function of habituation time, troop, time of day, and habitat features. We also model the relationship between each of the anti‐predator behaviors and ground‐use, exploring whether they predict greater terrestriality in the baboons.

**Results:**

In both troops, vocalization rates and observer‐directed vigilance declined with cumulative exposure to observers, but were heightened later in the day and in denser habitat types. We found that terrestrial activity was negatively related to levels of both vocalizations and observer‐directed vigilance.

**Discussion:**

This study provides a quantitative assessment of the impact of human observation on primate behavior and highlights environmental variables that influence anti‐predator behaviors, perhaps indicating heightened perception of risk. The relationship between perceived risk and terrestriality is significant for understanding the evolution of this rare trait in primates.

## Graphical abstract

- Habituation was used to investigate landscapes of fear in baboons.
- Antipredator behaviors—vocalizations and vigilance—declined with cumulative exposure to observers and were also affected by habitat structure and time of day.
- Both vocalization rates and levels of observer‐directed vigilance were negatively associated with baboons' use of the ground.

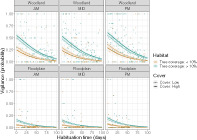

## 1 INTRODUCTION

Habituation is the period and process by which researchers accustom focal individuals or groups of wild animals to their presence, until the animals treat the observers as neutral stimuli in their habitat (Tutin & Fernandez, 1991). Although common practice in ethology, habituation is often viewed simply as a means to an end; namely, the collection of naturalistic data. Details about the process are thus underreported and studies rarely quantify the time taken for specific effects of observer presence on animal behavior to either plateau or disappear (Allan et al., 2020; Samuni et al., 2014; Williamson & Feistner, 2003).

Amongst primates, common initial reactions to the presence of humans include vocalizations, vigilance, threatening displays, and retreat, but the intensity of such responses decreases across repeated encounters with observers (Jack et al., 2008; Johns, 1996; McLennan & Hill, 2010; Schaller, 1962; Williamson & Feistner, 2003). The few published accounts of habituation indicate that great apes take at least 2–5 years to habituate to human presence (Williamson & Feistner, 2003), whilst “opportunistic” generalist taxa, such as *Papio* and *Macaca*, are thought to habituate to researchers comparatively quickly. However, even within the *Papio* genus there have been a range of habituation times reported—from 5 months to 2 years (Barton & Whiten, 1993; Cowlishaw, 1997b; Kummer, 1997; Noser & Byrne, 2007). This is convoluted further by the lack of a clear working definition for “habituated” primates, and the range in strategies and intensity of habituation efforts. Additionally, a recent study highlights that habituation does not occur at the same rate across groups but is influenced by each individual's tolerance of human presence (Allan et al., 2020).

Greater use of quantitative measures of habituation is thus needed to account for potential observer effects in behavioral studies, and to facilitate standardized comparison of behavioral and fitness data across studies and field sites. This is because animals may forego fitness‐enhancing activities such as foraging, mate‐seeking, or grooming to engage in observer‐directed behaviors such as vocalization or vigilance (Frid & Dill, 2002).

Human presence has also been linked to both proximate and more long‐lasting changes in primates' substrate use. Unhabituated primates often retreat from terrestrial observers into—or higher into—trees (Johns, 1996; van Schaik et al., 1983), and comparisons across field studies demonstrate longer‐term effects: habituated groups of primates exhibit more frequent and varied use of the ground than less habituated groups of the same species (*Ateles* spp.: Campbell et al., 2005; *Brachyteles hypoxanthus*: Mourthé et al., 2007). At its extreme, habituation shields primates from exposure to predators who avoid humans (e.g., leopards: Isbell & Young, 1993), which can inflate primate activity on or near the ground when humans are present (*Cercopithecus mitis erythrarcus*: Nowak et al., 2014). These examples highlight why observer‐effects—on unhabituated through to well‐habituated animals—must be understood, quantified, and incorporated into analyses of naturalistic behavior.

Quantitative monitoring of habituation also sheds light on adaptations to risk more generally. This is because predators evoke responses, trade‐offs, and changes to substrate‐use in primates, similar to those described above (Campbell et al., 2005; Cowlishaw, 1997b; Lima, 1998; Mourthé et al., 2007; Souza‐Alves et al., 2019). The term “landscape of fear” (Laundré et al., 2001) has been widely adopted in the study of predator–prey dynamics but its definition has remained ambiguous, largely due to conflation of patterns of actual risk, perceived risk, and behavioral responses to perceived risk. This ambiguity results in the use of unsuitable proxies and circular inferences about risk and response, and inhibits nuanced exploration of the mechanisms linking the two (Gaynor et al., 2019). Landscapes of fear have been defined variously as (1) the mapped physical environment, (2) spatial variation in actual predation risk, or (3) spatial patterns in antipredator behaviors. Gaynor et al. (2019) propose a framework in which these are three distinct, measurable landscapes, related in nonlinear ways and linked by the true landscape of fear: a fourth distinct layer representing *spatial variation in risk perception*.

These are important distinctions for exploring how ecological factors mediate predator–prey dynamics. For instance, habitat structure is reported to affect risk‐invoked behaviors in many prey species (Atuo & O'Connell, 2017; Gorini et al., 2011), including primates (Coleman & Hill, 2014; Enstam & Isbell, 2002, 2004; Hill & Weingrill, 2007). However, habitat could influence the cognitive experience of risk perception in several different ways. First, due to predators' habitat preferences or hunting techniques (ambush or cursorial), encounters between predators and prey are more likely in certain habitats and at certain times. Thus, prey might perceive greater risk in these areas and times, based on past experience. Second, habitat features such as low visibility reduce the probability of detecting predators, perhaps heightening perceived risk in certain habitats *regardless* of actual predator presence. And third, habitat features such as tree density might either aid or prevent primates escaping from predators, with these opportunities for risk mitigation altering the level of perceived risk that they experience (Hill & Weingrill, 2007).

For example, baboons' reported avoidance of areas with denser vegetation (Cowlishaw, 1997b; Hill & Weingrill, 2007; Rasmussen, 1983) might demonstrate a straightforward relationship between actual predation risk and prey response if predators are more prevalent or likely to attack in such habitats. However, even if predators are not actually present or concentrated in tall grass or wooded vegetation, it is argued that primates display heightened antipredator behaviors in such areas because reduced visibility increases the perception of risk in denser habitats (Altmann & Altmann, 1970; Cowlishaw, 1997b; Hill & Weingrill, 2007; Rasmussen, 1983). On the other hand, trees can provide refuge from predation and vantage points from which to detect terrestrial predators. Patas monkeys (*Erythrocebus patas*) were found to use taller‐than‐average trees to scan their surroundings and, despite predators being present across the landscape, made most successful predator detections from within these “tall habitats” (Enstam & Isbell, 2004).

Furthermore, the same risk stimulus—a mammalian predator alarm call—has been shown to evoke different antipredator responses depending on the habitat structure in which it is heard during playback experiments. Vervet monkeys (*Chlorocebus pygerythrus*) switched between arboreal and terrestrial escape strategies when they heard the same call in different environments (Enstam & Isbell, 2002). This behavioral flexibility is also demonstrated in a study where vervet monkeys reduced their levels of vigilance in areas that became more open after a fire (Jaffe & Isbell, 2009). These examples highlight the varied and nonlinear effects that vegetation might have on risk perception, and demonstrate that primates employ a range of flexible and compensatory behaviors to minimize their exposure to risk in the environment.

Measuring variation in actual predation risk across an environment is difficult, due to the elusiveness of carnivores, and the fact that their distribution and density across the landscape changes with time of day, season, and availability of prey and water. This makes it hard to untangle the contributing roles of actual risk and external ecological factors to prey animals' perception of risk. Habituation provides a quasi‐experimental setting in which focal animals are repeatedly exposed to the same risk stimulus (the presence of human observers) across time and habitat types. This provides an opportunity to elucidate the proximate effects of risk on behavior, as well as mediating effects of temporal and ecological factors on this relationship (Cowlishaw, 1997a; Frid & Dill, 2002; van Schaik et al., 1983).

Based on the assumption that perceived risk of humans declines with habituation, our study measures changes in (a) vocalization rates and (b) observer‐directed vigilance during primate habituation, and tests how each of these response variables is affected by ecological and temporal factors. We also test whether prevalence of these anti‐predator behaviors predicts levels of terrestrial behavior in baboons.

## 2 METHODS

### 2.1 Study site and species

Gorongosa National Park (−18.96°, 34.36°), Mozambique, lies at the southern end of the East African Rift System (EARS). It is composed of approximately 4000 km^2^ of heterogeneous habitats (Bouley et al., 2018; Stalmans & Beilfuss, 2008; Tinley, 1977) and is inhabited by over 200 troops of chacma baboons (Stalmans et al., 2018). Two focal troops were followed during this study: the Woodland Troop (WT) and the Floodplain Troop (FT), composed of 88 and 37 individuals respectively, based on counts at the end of the study period. As the names suggest, WT ranges predominantly in an *Acacia‐Combretum* savannah woodland, while FT ranges predominantly in floodplain grasslands, although they return to riverine sleep sites with more tree coverage. The baboons forage on a range of fruits, seeds, and foliage, as well as grass, rhizomes, and insects. Occasionally, individuals in both troops were also observed hunting and eating small vertebrates such as young antelope, warthogs, and ducklings.

Civil war in Mozambique between 1977 and 1992 decimated wildlife in the park; populations of large herbivorous mammals declined by >90%, and apex predators were almost completely lost from the landscape (Daskin et al., 2016). Before the war, leopard (*Panthera pardus*) and lion (*Panthera leo*) were abundant in the park, and wild dog (*Lycaon pictus*) and spotted hyena (*Crocuta crocuta*) were present in smaller numbers (Tinley, 1977). A small and slowly growing lion population survived the war (Bouley et al., 2018) but the other three carnivore species were not seen in the park until a single leopard sighting in April 2018, and the reintroduction of a pack of wild dogs to the park in June 2018 (Atkins et al., 2019).

Reduced predation pressure since the war has facilitated rapid growth of several mammal populations, including baboons (Atkins et al., 2019), and the troops in this study have had limited exposure to large mammalian carnivores. As this study marks the first primatological research in the park, the troops had also experienced minimal exposure to humans—especially humans on foot and off‐road—prior to the start of our data collection.

### 2.2 Data collection

We collected data during day follows in which each focal troop was followed by one researcher and one park ranger between the hours of 06:00 and 18:00 (although logistical constraints often limited this period to between 07:00 and 17:00, especially for FT). These follows began in April 2018 for WT and May 2018 for FT, and ended in November 2018 for both troops due to park closure in the wet season. Throughout follows, we attempted to remain as close to the troops as possible without eliciting obvious fleeing behavior. We used handheld GPS devices (Garmin Oregon 700 and Garmin Map64) to record tracks of the daily follow routes, and customized applications on Apple iPads to collect time‐stamped activity and habitat data.

The Animal Behavior Pro application (University of Kent, 2012) was used to conduct all‐occurrence vocalization sampling for 5 min every 30 min throughout the day. We logged every vocalization heard during the sample as a “Bark,” “Grunt,” “Copulation Call,” or “Contact Call.” As the clearest indicators of external risk (Fischer et al., 2001), only barks are included in the analyses below. There is a risk that some contact calls might have been included in the “bark” category, as they are not always easy to distinguish from one another, and acoustic analyses suggest there is a graded variation of vocalizations between tonal contact calls and harsher alarm barks (Fischer et al., 2001). While we expect human presence to have an effect on alarm barks over time (declining with habituation), we would not expect it to influence the frequency of contact calls between baboons. However, potential impacts of both habitat and time of day on contact calls will be addressed in the discussion. To account for troop size, vocalization data from WT and FT were divided by 80 and 30, respectively, producing troop‐adjusted vocalization rates.

The Animal Observer application (Dian Fossey Gorilla Fund International, 2012) was used to conduct activity scan samples every 30 min during follows until the September 14, 2018 and every 15 min after that date. As our analyses examine the probability of a behavior occurring per scan, this change in frequency should not affect our results. Scans were always conducted from left to right to minimize the chances of bias toward attention‐catching behaviors, and were conducted within a 5‐min limit. During scans, we logged the activity, vigilance‐state, and position (on or above the ground) of every monkey in view of the observer. Defining vigilance has proved problematic in primatology (Allan & Hill, 2018). To reduce ambiguity, vigilance was coded at the individual level as “looking directly at the observer during scan”, or “not looking directly at the observer during scan”. Individuals for whom vigilance‐state was unknown were excluded from the analyses below. Terrestriality was coded according to the position of each individual baboon during scans; those on the ground were coded as “terrestrial,” and those positioned on raised objects (such as rocks or fallen logs) or in trees were coded as “not terrestrial.”

**FIGURE 1:**
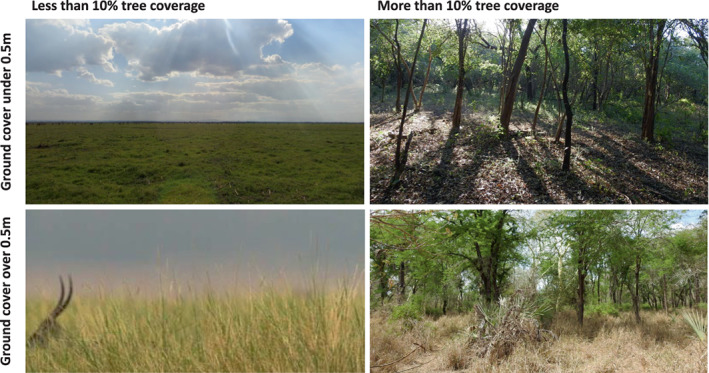
Examples of different habitat type combinations within the home ranges of the focal baboon troops

Habitat type and average height of ground cover were also recorded at the start of each scan, using the same application. For the analyses below, habitat type is coded as “More than 10% tree coverage,” “Less than 10% tree coverage,” or “Water.” The 10% tree coverage threshold was chosen to distinguish habitats in which the lack of trees is likely to dictate terrestriality. “Water” includes large pans or riverbanks and was included as a separate habitat type due to increased vulnerability of baboons whilst drinking and the potential risks of encountering other animals at major water sources. Estimations of the height of ground cover were coded as “Low” (under 0.5 m tall) or “High” (over 0.5 m tall), based on the approximate shoulder height of a female chacma baboon, to account for potential effects of ground cover on visibility. Examples of habitat type combinations are shown in Figure 1. Time‐stamped data were coded categorically as “AM” (06:00–09:59), “MID” (10:00–13:59), or “PM” (14:00–17:59).

### 2.3 Statistical analyses

We began by running generalized linear models (GLMs) to determine whether (a) vocalization rates and (b) the probability of observer‐directed vigilance changed over cumulative habituation time, measured in full follow days (>6 h data collection). We then tested whether these antipredator behaviors were significantly related to substrate‐use by baboons. All models were fitted in R v4.0.2 and we used likelihood ratio tests (LRTs) and the drop1 function from the “stats” package to select models (R Core Team, 2020). Maps and graphs were produced using the “sf” (Pebesma, 2018), “ggmap” (Kahle & Wickham, 2013), and “ggplot2” (Wickham, 2009) packages in R.

### 2.4 Vocalizations and vigilance models

#### 2.4.1 Vocalizations

Due to zero‐inflation in the vocalization data we used a hurdle model to explore changes in vocalization rates over habituation time. First, we fitted a GLM with a binomial error distribution and a logit link function to model binary data, classified as “at least one bark” (1) or “no barks” (0) during a scan. Then we fitted a GLM with a gamma distribution and a log link function to all non‐zero data to model bark rate as a continuous response. Predictions from each model were multiplied together to fit the final model.

#### 2.4.2 Observer‐directed vigilance

We used a binomial GLM with a logit link function to model the occurrence of observer‐directed vigilance over habituation time. As described above, this was coded as a binary variable, with individuals classified as “looking directly at the observer during scan” (1), or “not looking directly at the observer during scan” (0).

#### 2.4.3 Terrestriality models

Using the response variables from the models described above, we ran binomial GLMs with logit link functions to model the effects of (a) vocalization rates and (b) the likelihood of observer‐directed vigilance, on occurrence of terrestrial behavior. Terrestriality was coded as a binary variable at the individual level; “terrestrial” (1) or “not terrestrial” (0).

#### 2.4.4 Fixed effects

To account for potential intertroop differences, we included “Troop” as a fixed variable in all models described above. We also included time of day (“Diel”), habitat type (“Habitat”), and height of ground cover (“Cover”) as fixed explanatory variables, with an interaction between Habitat and Cover as both are vegetation features. All were chosen for their ecological significance in mediating anti‐predator responses.

### 2.5 Ethical note

This work was carried out with ethical clearance from Oxford University (APA/1/5/ACER/23Jan2018) and from the Ministry of Tourism and the Gorongosa Restoration Project in Mozambique (permit numbers PNG/DSCi/C114/2018 and PNG/DSCi/C93/2018). All data collection was observational in the troops' natural habitats and researchers did not come into contact with any of the animals.

## 3 RESULTS

**FIGURE 2:**
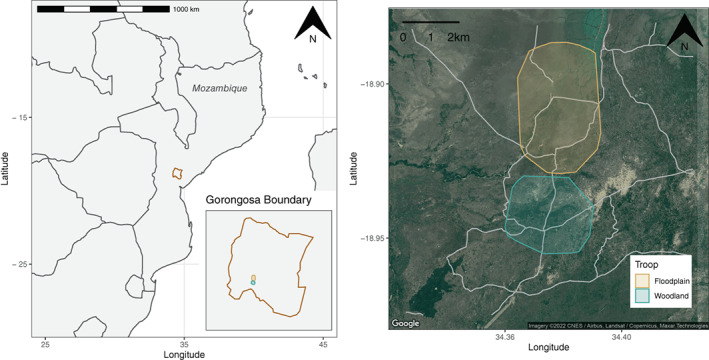
Map showing the location of Gorongosa National Park in Mozambique (left) with home ranges of the two focal troops highlighted within the park (inset) and shown as 95% minimum convex polygons overlaid upon roads (gray lines) and satellite imagery of vegetation within the park (right)

Between April and November 2018, WT was followed for a total of 921 h, distributed over 106 full follow days (average length: 8.64 h) and 14 partial follow days (<6 h). FT was followed for a total of 576.5 h, distributed over 63 full follow days (average length: 7.98 h) and 22 partial follow days. The mean break between follows was 2.47 days for WT (range: 1–19 days) and 3.26 days for FT (range: 1–29 days). WT ranged within a total area of 8.7 km^2^, predominantly in areas habitats with high tree coverage (72% of scans were conducted in habitats with more than 10% tree coverage, 9% in areas with less than 10% tree coverage, and 19% at major water sources). FT ranged within a total of 15.6 km^2^, predominantly within open habitats (67% of scans were conducted in habitats with less than 10% tree coverage, 24% in habitats with more than 10% tree coverage, and 9% at major water sources). It should be noted that FT significantly shifted their ranging patterns halfway through the field season, accounting for the greater overall size of their range. The reasons for this shift are currently under investigation. The troops' home ranges are shown in Figure 2.

### 3.1 Vocalizations and vigilance models

Likelihood Ratio Tests provide very strong evidence that Habituation time (Day Number) was a significant predictor of both the probability and rate of vocalizations, and of the probability of observer‐directed vigilance during this study. Of the fixed variables, there is strong evidence that Troop and Diel affected response variables across all three models, and that an interaction between the vegetation variables Habitat and Cover affected the probability of vocalizations and vigilance, but not the rate of vocalizations. These results are summarized in Table 1.

**TABLE 1:** Results of likelihood ratio tests for GLMs modeling (A) vocalization (i) probability and (ii) rates, and (B) observer‐directed vigilance probability over habituation time, and across troops, habitat types, and times of day.

	(A) Vocalization models				(B) Vigilance model	
	(i) Binomial		(ii) Gamma		Binomial	
	LRT	*p*	AIC scaled dev.	*p*	LRT	*p*
Day Number	65.97	< 0.001***	128.13	< 0.001***	680.70	< 0.001***
Troop	412.30	< 0.001***	7.34	0.01**	540.12	< 0.001***
Diel	21.18	< 0.001***	9.93	0.01**	27.30	< 0.001***
Habitat*Cover	8.31	0.02*	1.14	0.57	52.51	< 0.001***

*Note*: Asterisks indicate statistical significance of *p*‐values (* = < 0.05, ** = < 0.01, *** = < 0.001).

#### 3.1.1 Results from vocalization hurdle model

**FIGURE 3:**
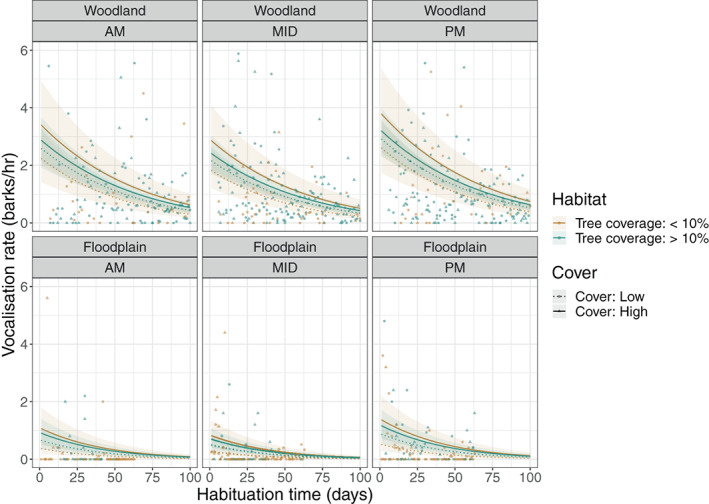
Changes in baboon vocalization rates over habituation time (measured in number of full day follows) for the woodland troop (top row) and floodplain troop (bottom row), grouped by time of day. Habitat types are differentiated by color, and ground cover by differently shaped data points and line types.

The results from the vocalization hurdle model, listed in Table 2, provide very strong evidence that both the probability and rate of vocalizations during scans decreased over habituation time, and were significantly lower in FT than WT. The binomial model results (Table 2A‐i demonstrate that vocalizations were more likely to occur later in the afternoon (PM) than at any other time of day and were less likely in habitats with less than 10% tree coverage than the other two habitat types. High ground cover increased the likelihood of vocalizations occurring, particularly in habitats with less than 10% tree coverage. The gamma model results (Table 2A‐ii show that vocalization rates decreased significantly over habituation time and were consistently lower in FT, but there is no evidence that any of the other fixed effects influenced vocalization rates. Vocalization data and results from the hurdle model are plotted in Figure 3.

**TABLE 2:** Results from GLMs showing the effects of habituation time and fixed variables on (A) (i) Probability of vocalizations, (ii) rates of vocalizations, and (B) probability of observer‐directed vigilance by baboons

GLM	Estimate	Std. error	*p*
*(A‐i) Vocalizations*: *Binomial*			
(Intercept)	1.81	0.17	< 0.001***
Day Number	−0.01	0.00	< 0.001***
Troop: Floodplain	−2.62	0.14	< 0.001***
Diel: MID	−0.18	0.12	0.13
Diel: PM	0.30	0.13	0.02*
Habitat: Open	−0.75	0.15	< 0.001***
Habitat: Water	−0.18	0.14	0.19
Cover: High	0.41	0.14	< 0.003**
Habitat: Open*Cover: High	0.73	0.25	< 0.004**
Habitat: Water*Cover: High	0.15	0.39	0.70
*(A‐ii) Vocalizations*: *Gamma*			
(Intercept)	1.12	0.11	< 0.001***
Day Number	−0.01	0.00	< 0.001***
Troop: Floodplain	−0.34	0.12	< 0.001***
Diel: MID	−0.15	0.09	0.09
Diel: PM	0.08	0.09	0.37
Habitat: Open	0.00	0.12	0.98
Habitat: Water	0.18	0.10	0.07
Cover: High	0.06	0.09	0.53
Habitat: Open*Cover: High	0.17	0.21	0.42
Habitat: Water*Cover: High	−0.23	0.38	0.54
*(B) Vigilance*			
(Intercept)	0.01	0.09	0.93
Day Number	−0.03	0.00	< 0.001***
Troop: Floodplain	−1.82	0.08	< 0.001***
Diel: MID	−0.06	0.07	0.35
Diel: PM	0.24	0.07	< 0.001***
Habitat: Open	−0.76	0.09	< 0.001***
Habitat: Water	−0.64	0.07	< 0.001***
Cover: High	0.23	0.08	0.004**
Habitat: Open*Cover: High	−0.56	0.21	0.01**
Habitat: Water*Cover: High	1.46	0.21	< 0.001***

*Note*: Asterisks indicate statistical significance of *p*‐values (* = < 0.05, ** = < 0.01, *** = < 0.001).

#### 3.1.2 Results from observer‐directed vigilance model

**FIGURE 4:**
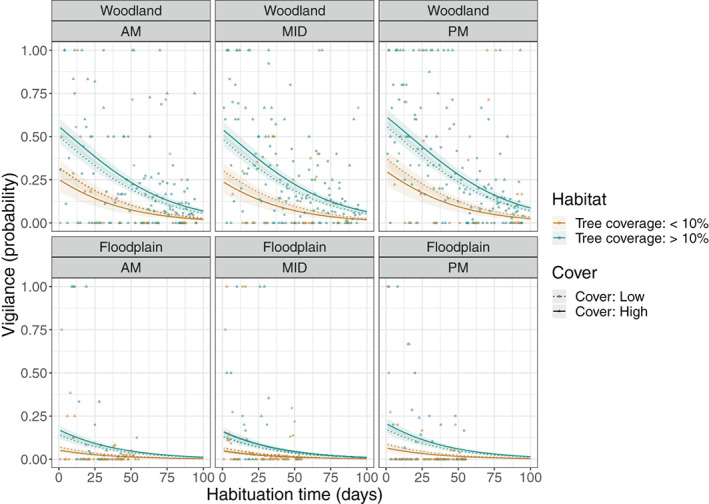
Changes in the probability of observer‐directed vigilance over habituation time (measured in number of full day follows) for the woodland troop (top row) and floodplain troop (bottom row), grouped by time of day. Habitat types are differentiated by color, and ground cover by differently shaped data points and line types.

The vigilance model results, also listed in Table 2 (model B), demonstrate very strong evidence that the probability of observer‐directed vigilance occurring during a scan decreased over habituation time and, as with vocalizations, was lower in FT than WT. The results provide strong evidence that observer‐directed vigilance was more likely to occur in the late afternoon and in habitats with more than 10% tree coverage than the other two habitat types. High ground cover increased the likelihood of observer‐directed vigilance. An interaction effect between habitat variables suggests that vigilance was most likely at water sources with high ground cover, and least likely in habitats with less than 10% tree coverage but high ground cover, compared to other habitat combinations. Vigilance data and results from this model are plotted in Figure 4.

### 3.2 Terrestriality models

Table 3 summarizes the results from two GLMs, run with (A) vocalization rate and (B) likelihood of observer‐directed vigilance as predictors of terrestriality. Both higher vocalization rates and levels of observer‐directed vigilance predict a lower likelihood of baboons being on the ground during a scan. Inclusion of fixed variables in the models highlights that FT was more terrestrial than WT, that terrestriality was highest in late afternoon, and that baboons were more terrestrial in habitats with less than 10% tree coverage and at water sources than in habitats with more than 10% tree coverage. High ground cover significantly reduced terrestriality, particularly at water sources and areas with less than 10% tree coverage.

**TABLE 3:** Results from GLMs using (A) vocalization rate, and (B) the likelihood of observer‐directed vigilance as predictors for the occurrence of terrestrial behavior in baboons during habituation

GLM	Estimate	Std. error	*p* Value
*(A) Vocalization rate as predictor*			
(Intercept)	0.68	0.06	<0.001***
Vocalization rate	−0.17	0.05	<0.001***
Troop: Floodplain	0.66	0.07	<0.001***
Diel: MID	−0.01	0.05	0.88
Diel: PM	0.43	0.06	<0.001***
Habitat: Open	2.21	0.09	<0.001***
Habitat: Water	2.00	0.06	<0.001***
Cover: High	−0.85	0.06	<0.001***
Habitat: Open*Cover: High	−0.72	0.13	<0.001***
Habitat: Water*Cover: High	−2.13	0.17	<0.001***
*(B) Vigilance as predictor*			
(Intercept)	0.60	0.06	<0.001***
Vigilance	−0.51	0.24	0.03*
Troop: Floodplain	0.72	0.06	<0.001***
Diel: MID	0.01	0.05	0.82
Diel: PM	0.41	0.06	<0.001***
Habitat: Open	2.21	0.09	<0.001***
Habitat: Water	1.96	0.07	<0.001***
Cover: High	−0.87	0.06	<0.001***
Habitat: Open*Cover: High	−0.77	0.13	<0.001***
Habitat: Water*Cover: High	−2.04	0.18	<0.001***

*Note*: Asterisks indicate statistical significance of *p*‐values (* = < 0.05, ** = < 0.01, *** = < 0.001).

## 4 DISCUSSION

We modeled the change in vocalization rates and levels of observer‐directed vigilance over several months of habituation of two troops of chacma baboons, demonstrating that both measures declined with cumulative exposure to observers. Both measures also varied predictably by troop, time of day, the openness of the environment and ground cover. Both vocalization rates and observer‐directed vigilance had a negative relationship with the likelihood of ground‐use by the baboons.

Such models can be used to explore whether, or when, habituation achieves its purpose of allowing focal animals to treat observers as neutral stimuli in their environment. For example, based on daily means from our study, the likelihood of observer‐directed vigilance dropped below 5% after 95 days in WT and after 44 days in FT. Although this threshold is somewhat arbitrary, recording and reporting such measures provides a standardized method for accounting for observer presence. By measuring the extent to which observers might be interfering with focal animals' normal activity patterns these indicators can be incorporated into analyses of behavioral data collected from different stages of habituation and from different study troops or sites. Such measurements could also serve as a reminder that observers might never be neutral features in study animals' environments. The growing use of remote sensing tools such as camera traps and activity loggers will shed light on the extent to which animals considered habituated to human observers are exhibiting “natural” behaviors.

Our results also highlight that, even between the two troops in this study, habituation appears to have occurred at quite different rates; both vocalization rates and observer‐directed vigilance were consistently lower in FT than WT throughout the study. There are several factors that might be at play here. WT is double the size of FT and it might have taken longer for all individuals in the bigger troop to be exposed to the observers. Furthermore, this study does not account for individual differences in tolerance to humans or risk, which could account for different rates of habituation both within and between troops (Allan et al., 2020). Alternatively, or additionally, difference in vocalizations and vigilance across the troops might reflect how habitat features in their respective home ranges influence their perceived risk of observers. Our results suggest that lower visibility in the predominantly closed habitats of WT increased antipredator behaviors in response to humans amongst these woodland‐dwelling baboons compared to those ranging on the floodplain.

This pattern is also reflected within the home range of each troop, with both vocalizations and vigilance more likely to occur in areas where vegetation structure—greater tree density or taller ground cover—reduces visibility. Compared with other vegetation combinations, areas with tree density less than 10% but high ground cover predicted a greater likelihood of vocalizations but lower levels of vigilance, perhaps highlighting how vegetation structure determines the most appropriate responses to perceived risk. When available, trees might serve as lookout points from which to detect and be vigilant of risk, but when they are unavailable, vocalizations might be used to alert others to risk or to prevent separation of the troop in areas of high ground cover (Byrne, 1981).

Our results show that temporal as well as spatial factors mediate risk responses. Across habitat types, baboon vocalizations and vigilance were more likely in the late afternoon than earlier in the day, suggesting that the perceived risk of observers was heightened at this time of day. This might signal an adaptation to avoiding risk from predominantly nocturnal predators. The extant carnivore guild (with the exception of cheetahs) hunt most successfully at night and in crepuscular hours (Treves & Palmqvist, 2007), and many baboon predation events occur at their sleeping sites, to which they would be traveling in the late afternoon (Cowlishaw, 1994; Isbell et al., 2018). The reduction of visibility in twilight hours might also contribute to increases in vocalizations and vigilance towards the end of the day.

It should also be noted that some of the vocalizations recorded in our “bark” sample might in fact have been contact calls rather than alarm barks, as these two types of vocalization are not always clearly distinguishable (Fischer et al., 2001). Whilst we attribute the clear decline in bark rates over habituation time to a reduction in baboons' perceived risk of researcher presence, it is possible that the influence of both habitat type and time of day on vocalization rates might be capturing some effects of those variables on contact calls. For example, baboons may need to make contact calls more frequently in closed environments or towards the evening as they congregate at their sleep sites, and this increase in vocalizations might have been recorded in our counts of barks. However, the fact that observer‐directed vigilance is similarly influenced by these variables provides an independent source of evidence for the effects of habitat and diel periods on anti‐predator behaviors.

Finally, we consider the relationship between antipredator behaviors and terrestriality. Baboons are a highly terrestrial species but—like most primates—rely on trees for refuge, for example by seeking out trees and cliffs as sleeping sites. Threat from large mammalian carnivores (or perceived threat from human observers) might therefore reduce baboons' time spent on the ground. Terrestriality is of course inextricably linked to habitat structure, and this is clear in the results from our analyses. The Floodplain Troop were found to be significantly more terrestrial than the Woodland Troop, and within each troop's home range the baboons were more likely to be on the ground in areas with minimal tree coverage and lower ground cover. They were also found to be more terrestrial in the late afternoon. This might be explained by baboons' tendency to forage for depletable resources, such as arboreal fruits, in the morning before feeding on less desirable but more abundant foods, such as seeds and grasses, later in the day (Noser & Byrne, 2007).

Beyond these predictable effects of habitat structure and possible consequences of daily routine, our analyses demonstrate that higher levels of antipredator behaviors—vocalizations and vigilance—predict lower occurrence of terrestriality in baboons. These results have implications for the study of both extant and extinct primate species. First, they imply that researchers may perceive a study group to be more arboreal than they really are before the effects of observer‐induced fear wear off. And at the other extreme, when primates are well‐habituated and observer presence keeps carnivores at bay, this shift in the landscape of fear might inflate the time focal primates spend on the ground. This again questions whether researchers can ever be considered neutral stimuli in their study animals' environments and emphasizes the need for incorporating quantified effects of observer‐presence into behavioral studies.

The second set of implications of these results concerns the evolution of terrestriality in certain primate species, including baboons and humans. Although it is common for primates to occasionally come to the ground, particularly during travel, very few non‐human species are considered terrestrial or even semiterrestrial (Galán‐Acedo et al., 2019). Modern humans are unique among primates in both their obligate terrestriality and bipedalism. There has been more focus on the evolution of bipedality than terrestriality in our lineage, but their origins are not necessarily linked (Harcourt‐Smith, 2010; Richmond et al., 2001).

Broad shifts in habitat structure from closed to more open environments have often been postulated as the primary driver of increasingly terrestrial adaptations in hominins and other African terrestrial primates (Dart, 1925, 1949; Harcourt‐Smith, 2010; Jablonski, 2005; Senut et al., 2018). In the case of hominins, this “Savannah Hypothesis” has become more nuanced as evidence has emerged that the earliest hominins inhabited areas with relatively high tree coverage (Bobe et al., 2020; White et al., 2009) and that adaptations to arboreality are retained in species of the *Australopithecus*, *Paranthropus*, and *Homo* lineages (Dunmore et al., 2020; Feuerriegel et al., 2017; Richmond et al., 2020; Ruff, 2009; Stern & Susman, 1983; Ward, 2002). Some researchers have suggested that modern human locomotion emerged in a series of distinct shifts from “occasional” to “facultative,” and then to “obligate” terrestrial bipedalism, each occurring in different ecological contexts and therefore influenced by separate selective forces (Harcourt‐Smith, 2010; Jablonski, 2005). Our study indicates that the landscape of fear should be considered amongst such forces. Alongside changing habitat structure, turnovers in the African carnivore guild would have altered the amount and distribution of risk across certain habitats, particularly as the abundance and diversity of carnivores declined from the Pliocene to the modern day (Treves & Palmqvist, 2007). Our results indicate that reduced risk from terrestrial predators and a consequent rise in time spent on the ground might have helped drive terrestrial traits to fixation in African terrestrial primates, including hominins.

## 5 CONCLUSION

In this study, we recorded chacma baboons' vocalizations and observer‐directed vigilance over several months to monitor changes in the frequency of their anti‐predator responses to humans during habituation, and possible effects on their terrestriality. Our results suggest that vegetation structures which reduce visibility increase perceived risk, and that baboons experience heightened perception of risk later in the day, which might be an adaptive response to avoiding predominantly nocturnal predators. Our results also indicate that, across habitats and times of day, higher levels of antipredator behaviors predict lower chances of baboons spending time on the ground, suggesting that perceived risk reduces terrestriality in baboons. A better understanding of the extent to which substrate‐use is determined by risk perception will be significant for incorporating the effects of human presence into studies of primate behavior, and for understanding the origins and evolution of terrestriality – a rare trait amongst primates.

## AUTHOR CONTRIBUTIONS

**Philippa Hammond:** Conceptualization (lead); data curation (lead); formal analysis (lead); investigation (equal); methodology (equal); project administration (equal); visualization (lead); writing – original draft (lead). **Lynn Lewis‐Bevan:** Conceptualization (equal); data curation (equal); investigation (equal); methodology (equal); software (equal); writing – review and editing (supporting). **Dora Biro:** Conceptualization (supporting); formal analysis (supporting); investigation (supporting); methodology (supporting); supervision (equal); writing – review and editing (supporting). **Susana Carvalho:** Conceptualization (supporting); formal analysis (supporting); investigation (supporting); methodology (supporting); project administration (equal); resources (equal); supervision (equal); writing – review and editing (equal).

## CONFLICT OF INTEREST

The authors declare no conflict of interest.

## Data Availability

The data collected and analyzed for this study are available from the corresponding author upon reasonable request.
